# Effect of autologous fibrin glue on seroma reduction after modified radical mastectomy for breast cancer: A randomized controlled trial^☆^

**DOI:** 10.1016/j.amsu.2021.01.083

**Published:** 2021-02-01

**Authors:** Mohammed Faisal, Sara Salem, Noha kamel, Haidi Abd -Elzaher, Ahmed Abo Bakr, Hamada Fathy

**Affiliations:** a Surgical Oncology Unit, Department of Surgery, Faculty of Medicine, Suez Canal University Hospital, Egypt; b Clinical Pathology Department, Faculty of Medicine, Suez Canal University, Ismailia, Egypt

**Keywords:** Breast cancer, Seroma, Fibrin glue, Autologous

## Abstract

**Introduction:**

Breast cancer stands out as the second most common cancer in the world with incidence 35.1% of all malignancies among females in Egypt. Fluid build-up after breast surgery is still the most annoying complication which leads to worse outcome. We aimed to evaluate whether autologous fibrin glue might lessen the formation of seroma following modified radical mastectomy.

**Methods:**

This was a randomized controlled trial designed to configure the effect of autologous fibrin glue given in the study group using the drain in comparison to a control group who received the drain only; seroma volume was calculated every 24 h. For all of the cases. The drains were removed when the daily drainage was less than 30 ml for 3 consecutive days.

**Results:**

We recruited 30 patients to each of the two groups. Age, pathology, breast cancer stage, number of lymph nodes and tumour size did not differ significantly between groups. A comparison of the median days to drain removal showed 8 days reduction in median days to drain removal compared in the intervention group (7 days) than the control (15 days). The patients in the fibrin glue group had a significantly lower cumulative drain output volume (mean ± SD of 505,6 ± 209,3 ml) than those in the control group (1674.1 ± 1 373,8 ml).

**Conclusions:**

Autologous fibrin glue significantly decrease seroma formation post-modified radical mastectomy.

## Highlights

• Seroma is the most annoying complication after breast cancer surgery.
• Fibrin glue is an adhesive agent has been associated with reduction in the fluid build-up post mastectomy.
• Autologous Fibrin Glue is easily prepared and safe agent.
• The usage of Autologous fibrin glue help in short hospital stay after mastectomy.
• This is the first randomized clinical intervention to use fibrin glue from autologous nature to reduce seroma formation after MRM.

## 1 Introduction

Breast cancer stands out as the second most common cancer in the world and is the commonest in women. The distribution varies significantly between continents from 27 in 100,000 in Central Africa and Eastern Asia to 96 per 100,000 in Western Europe [1].

Seroma is a prevalent and annoying problem post breast and lymph nodes removal leading to the majority of complications that happens, for instance, wound infection, skin flap necrosis, wound dehiscence, and lymphedema. Because of the potentially severe effects of seroma, several approaches have been described to obliterate the dead space aiming at reduction of seroma formation; for example, using compression bandage, suction drains, flap fixation with sutures, utilization of ultrasound scalpel or laser scalpel in dissection, sclerotherapy, immobilization from the affected arm, topical use of tranexamic acid and fibrin glue application [[2], [3], [4], [5]].

Fibrin glue, light-activated fibrin sealant, and transdermal photopolymerized adhesive have been associated with reduction in the fluid build-up post mastectomy through various studies [[6], [7], [8], [9]]. Fibrin glue acts by lessening the number of disrupted lymphatics and minute vessels during axillary dissection hence resulting in lowering of seroma output [6,10] and provide haemostasis by controlling the hematomas which may delay wound healing. In addition, the fibrin glue has been recommended to interfere with the broken tissues during surgery, promoting the emergence of fibroblasts so enhancing closure of the wound through blockage of the dead spaces by tissue adhesion [5,11].

However, conflicting results have been reported regarding whether the fibrin glue plays a role in lowering the generation of seroma after breast cancer surgery or not [[12], [13], [14]].

Therefore, we aimed to reduce seroma formation after modified radical mastectomy (MRM) by using autologous fibrin glue, which has the advantage of being safe and inexpensive.

## 2 Methods

This research was a randomized controlled trial that was carried out at the surgery departments of Suez Canal college Hospital from July 2017 to december 2018. The study was reviewed by our research ethics committee in the Faculty of Medicine of Suez Canal University at its meeting on June 11, 2017. The study adhered to CONSORT guideline.

### 2.1 Patients

In the current work, sixty consecutive patients who were diagnosed with breast cancer and treated by MRM were recruited.

Newly diagnosed breast cancer stage I or II, who had no previous surgery on the axillary lymphatic system on the same side, no previous radiotherapy, nor corticosteroid treatment were included.

Exclusion criteria were (1) patients on systemic steroid or anticoagulants. (2) Having coagulation disorder, uncontrolled diabetes, advanced liver disease or chronic kidney disease. (3) Patients with history of chest radiation (as it can increase risk of seroma formation). (4) Patients arranged for breast reconstruction at the same session.

Age not less than 18 years (Eligibility for autologous transfusion).

### 2.2 Autologous fibrin glue preparation

The fibrin glue was prepared at Suez Canal University Hospital blood bank. All recruited patients were eligible for autologous blood donation. Three days prior to surgery, approximately 450 mL were obtained in triple blood bags containing 70 mL CPDA-1 (purchased from JMS, Singapore). The plasma was separated from the red blood cells within 2 h by centrifugation (Centrifuge: Thermo SorvallTM RC 12BP, Thermo Fisher Scientific, USA) at 2200×*g* for 10 min. The plasma was frozen and stored for 48 h at −40 °C. Then, fresh frozen plasma was thawed at 4 °C for 8 h before centrifugation at 2200×*g* for 10 min and the supernatant plasma was transported to the other satellite bag. The cryoprecipitate was suspended in 15 ml of plasma. Seven millilitres of fibrinogen and factor XIII (which is a fibrin stabilizing factor) containing precipitate were drawn into a syringe.

An hour before the operation. We used autologous thrombin obtained by drawing 14 ml of blood from the patient in plain tubes, which were left 30 min until clotting occurred and then centrifuged at 1000×*g* for 12 min; then the supernatant was collected (About 7 ml), which represents autologous thrombin [15]. Then, we added the cryoprecipitate in equal volume to the thrombin. Finally, we added 2 ml calcium gluconate (50 mg/mL calcium gluconate monohydrate) just before 2 min of application into the flaps.

### 2.3 Randomization

The patients were randomly allocated to either the intervention or control group. Once the patient consented to enter the trial, each patient was randomly assigned using random function of the Microsoft Excel programme. Sealed opaque envelopes were given to the surgeon. Patients were equally allocated at a ratio of 1:1 to either the intervention or control group.

### 2.4 Intervention

**Fig. 1:**
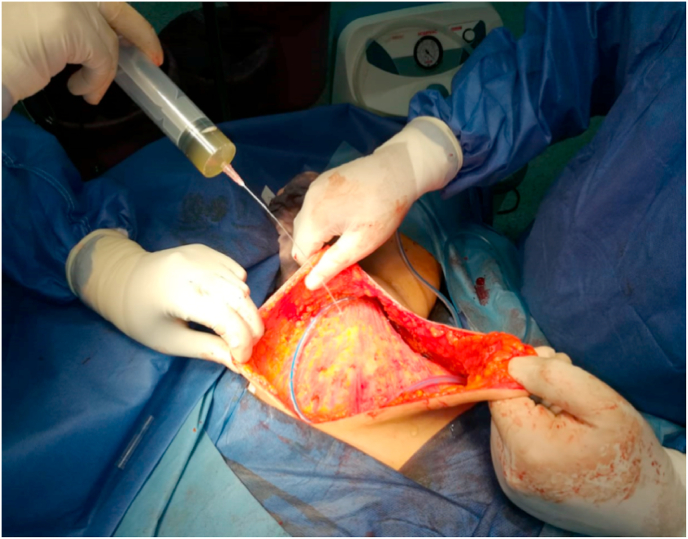
Application of the fibrin glue after MRM.

All patients underwent MRM. The same technique was followed in both groups; the use of electrocautery was minimized as much as possible, and meticulous haemostasis was performed. After mastectomy, the intervention cases received fibrin glue plus drain insertion before wound closure. The prepared glue mixture was sprayed after the field had dried completely; 8 ml were applied to the dead space under the skin flaps, and the other 8 ml were applied to the axillary bed (Fig. 1).

After spraying the mixture, we exerted gentle pressure for at least 5 min over the flaps and axilla and then closed the wound in layers as rapidly as possible. Last, we placed a small compressing cotton pad in the axilla. In the control group, we performed MRM using the same technique with meticulous haemostasis but closed the flaps immediately after inserting the drains (In both groups, we inserted two separate suction drains one at the axilla, and the other under skin flaps).

In both groups, we removed the drains once the volume of drained fluid was less than or equal to 30 cm3 per day, for 3 successive days.

### 2.5 Follow up

All patients were discharged 24 h after surgery with drains. A card was given to each patient at the time of discharge to record the drain volume at home daily at a specified time of the day, and the volume was measured after placing the bottle on a flat surface. Patients were followed up in the outpatient clinic until drain removal with instructions to return to the clinic early if the drain bottle was filled up, if leakage from around the drain was encountered, or if the drain bottle's vacuum was lost. The drains were removed when the drainage volume was less than 30 ml over 24 h for 2 successive days.

### 2.6 Outcome measures

The major factor assessed in the trial was the amount of drained fluid in the postoperative period; the secondary outcome was the duration took to remove the drain. In addition, we recorded the number of excised lymph nodes and the pathology results.

### 2.7 Statistical analysis

The gathered information was examined using IBM Statistical Package for Social Sciences software (SPSS), 21st edition. Continuous data were expressed as mean ± standard deviation and categorical data as frequencies and percentages. When data were tested for normality, they were not normally distributed, therefore, Mann Whiney *U* test and Kruskal Wallis test were used to compare continuous variables between different groups. Fisher's exact test and chi-square test were used for statistical analysis of categorical variables. Time-to-drain removal was analysed with survival analysis. Survival function was presented as Kaplan Meier curve, while the log rank test was used to test for the statistical significance of the difference in survival distribution between the intervention group and its control. For all tests, a probability value of less than 0.05 was considered statistically significant.

## 3 Result

Sixty Patients were enrolled and at random assigned to either the intervention (n = 30) or control group (n = 30). Their baseline characteristics (age, pathology type, and tumour stage and breast weight) are represented in Table 1. The mean age was 53 ± 4.7 years in the control group and 50 ± 3.7 years in the intervention group, without any significant record distinction among the particular groups. The majority of the patients had invasive ductal carcinoma (93.3%); 2 had stage I lesions, and 58 had stage II lesions (Table 1).

**Table 1:** Baseline characteristics of the patients (n = 60).

Variables	Total (n = 60)	Patients treated with fibrin glue (n = 30)	Patients not treated with fibrin glue (n = 30)	*p*-value
Age (years, mean ± SD)	51.4 ± 5.6	50.1 ± 3.7	53 ± 4.7	0.24^a^
***Pathology,*** (number (%))				
Invasive ductal carcinoma	56 (93.3)	26 (86.7)	30 (100)	0.112^b^
Others^a^	4 (6.7)	4 (13.3)	0 (0)	
***Stage*** (number (%))				
I	2 (3.3)	2 (6.7)	0 (0)	0.15^b^
II	58 (96.7)	28 (93.3)	30 (30)	
***Breast weight*** (number (%))				
500–1000 g	17 (28.3)	7 (23.3)	10 (33.3)	0.054^b^
>1000 - <3000 g	37 (61.7)	17 (56.7)	20 (66.7)	
>3000 g	6 (10)	6 (20)	0 (0)	

^a^*P*-value is based on the Mann-Whitney *U* test. Statistical significance at *p* < 0.05.

^b^*P*-values are based on the Fisher exact test. Statistical significance at *p* < 0.05.

a Others: Invasive lobular carcinoma, Paget's disease of the breast.

The entire amount of seroma formation in the group who received fibrin glue was 505,6 ± 209,3 ml, while in the control group it was 1 674,1 ± 1 373,8 ml (*p* < 0,001) (Table 2).

**Table 2:** Comparison of lymph node count, tumour size, volume of fluid, period till drain removal and in patients treated with and without fibrin glue (n = 60).

Variables	Total (n = 60)	Patients treated with fibrin glue (n = 30)	Patients not treated with fibrin glue (n = 30)	*p*-value
Lymph node count				
≤ 15	14 (23.3)	7 (23)	7 (23)	0.191^a^
15-25	34 (56.7)	20 (66.7)	14 (46.7)	
> 25	12 (20)	10 (33.3)	2 (6.7)	
**Tumour size**				0.622^a^
<3 cm	18 (30)	10 (33.3)	8 (26.7)	
3–5 cm	38 (63.3)	20 (66.6)	18 (60)	
> 5 cm	4 (6.66)	2 (6.66)	2 (6.66)	
**Total volume of fluid (ml)**		505.6 ± 209.3	1674.1 ± 1373.8	<0.001*
**Period till drain removal (days)**		8.5 (7–10)	15 (10–23)	0.01*

^a^ P-values are based on the chi-square test. Statistical significance at *p* < 0.05.

* P-value is based on the Mann-Whitney *U* test. Statistical significance at *p* < 0.05.

A comparison of the period till the drain removal between the studied cases showed that the patients who were treated using fibrin glue after surgery had a significantly shorter period (8.5 (range 7–10) days) than those who were not treated using fibrin glue (15 (10–23) days) (*p* = 0.01) (Table 2).

**Fig. 2:**
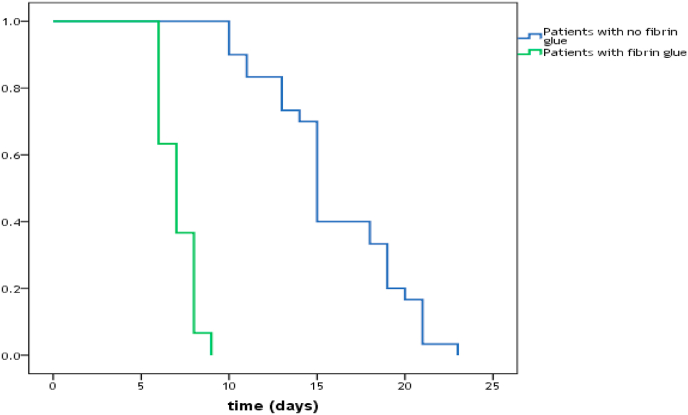
Time to drain removal in modified radical mastectomy patients in both groups.

The usage of fibrin glue after mastectomy and axillary clearance showed 8 days reduction in median days to drain removal compared with the group who did not use fibrin glue (log-rank p-value = 0.01) (Fig. 2).

Many factors other than fibrin glue can affect total volume of fluid build-up such as amount of excised lymph nodes, tumour size and breast weight, in the present study we found non-significant difference between patients from both groups regarding those factors.

**Fig. 3:**
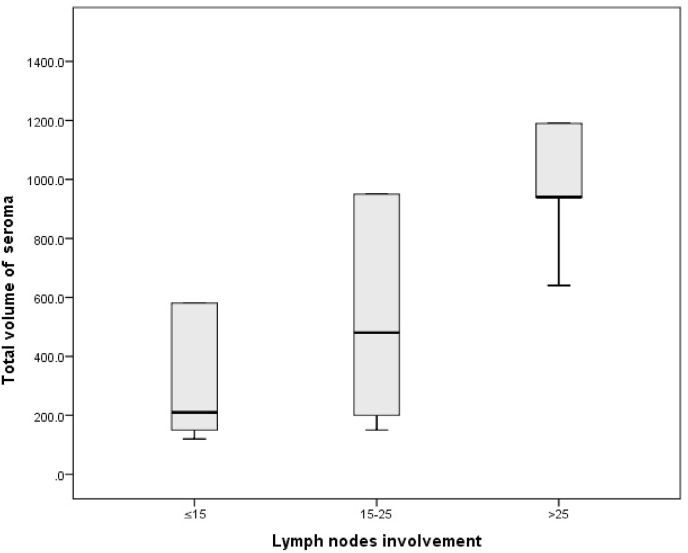
Association between total volume of seroma and lymph nodes involvement.

In this study we found that higher number of excised lymph nodes were associated with significantly more seroma volume (*p* < 0.001). On applying post hoc test, it was found that patients with more than 25 excised lymph nodes had significantly more seroma volume than all other groups (*p* < 0.001), and patients with 15–25 excised lymph nodes had significantly more seroma volume than those with less than 15 excised lymph nodes (*p* < 0.001) (Fig. 3).

Additionally, higher tumour size was associated with significantly more seroma volume (*p* < 0.001). On applying post hoc test, it was found that cases with tumour size >5 cm had significantly more seroma volume than tumour size 3–5 cm and ≤3 cm (*p* < 0.001), and patients with tumour size 3–5 cm had significantly more seroma volume than those with tumour size ≤ 3 cm (*p* < 0.001) (Table 2).

Moreover, patients with breast size of more than 1000 to less than 3000 gm and those with breast size more than 3000 gm had significantly more seroma volume than those with size between 500 and 1000 gm (*p* < 0.001) (Table 2).

## 4 Discussion

A documented method for seroma reduction post breast excision has not yet been established. So, we aimed to reduce seroma formation by safe, cheap and easily brought method through applying autologous fibrin glue.

In the present study, we found a significant decrease in the total volume of the fluid in the group who received fibrin glue compared to the control. This finding is in accordance with the results documented by other authors who used commercial fibrin glue in their interventions [5,[7], [8], [9]].

However, conflicting results were found by other authors, who reported inconclusive results or even harmful effects of fibrin glue which were explained by the presence of drains that may interfere with the maturation of the fibrin clot and the blockage of the lymphatic vessels [[16], [17], [18]]. However, there are important variations between those studies and ours. **Cha** and his colleagues applied fibrin glue following a different surgical treatment, an instantaneous breast renovation employing a latissimus dorsi myocutaneous flap [13]. While, **Burak** and co-authors [12] used only bovine thrombin which constitutes one element of fibrin glue, which might explain why they were unsuccessful to acquire a significant outcome, and similar outcome was reported by **Cipolla** and colleagues [6] in their study. Using fibrin glue in a low or unsuitable concentration, using bovine thrombin and fibrinogen, or using only one of these two components might reduce the effectiveness of fibrin glue. Therefore, we opted to utilize autologous fibrinogen and thrombin in our fibrin glue preparation. Additionally, we closed the wound rapidly after the fibrin glue was applied because it appears to be really vital that you take the most from the sealing effect and adhesive strength from the glue. When the wound is quickly closed after using glue, the wound surfaces can effectively bond with the glue polymerization period, however, if there's a delay, polymerization within the glue can happen before tissue adherence is completed. The polymerized glue may hinder rather than allowing adhesion effectively, being a barrier between opposing tissue surfaces [10,19]. In the present study, we took great care to avoid this condition by mildly compressing the wounds immediately after spraying the fibrin glue for at least 5 min, and then closing the wounds as rapidly as possible.

Using fibrin glue reduced the drainage duration in breast cancer patients undergoing level II or III axillary dissection [20]. In contrast, a study by **Ulusoy** and co-authors [18] found no obvious change in the daily output, drain removal time, seroma formation frequency between the two groups.

Regarding the factors associated with the volume of the seroma, we found that lower preoperative breast weight, lower tumour stage, lesser number of excised lymph nodes, and smaller tumour size were all associated with smaller amounts of fluid. This agrees with a previous study [17] but is contrary to what was reported by other studies who found that tumour size and location, histological type, disease site and specimen weight were not associated with increased seroma formation [21,22].

Finally, the utilization of fibrin glue led to a median of 8 days reduction in the time required to drain removal. This can be explained by the effectiveness of autologous fibrin glue in lessening the amount of seroma, and therefore reducing the period needed to drain removal.

To our knowledge, the current research represents the first work to use fibrin glue from autologous nature to reduce seroma formation after Modified Radical Mastectomy for breast cancer as a randomized clinical intervention, which makes our finding unique even though they are borderline significant.

The limitations of this study include small sample size, and short follow up duration. All the patients treated with autologous fibrin glue had no seroma collected after drain removal in opposite to what happened with most of the control group; however, we did not follow up for longer period after drain removal.

## 5 Conclusion

The present study supports the utilization of autologous fibrin glue in order to lessen post-modified radical mastectomy seroma. Further larger prospective studies and follow up for longer period after drain removal are recommended.

## Ethical approval and consent to participate

All procedures performed in our study involving human participants was in accordance with the ethical standards of the institutional and/or national research committee and with the 1964 Helsinki declaration and its later amendments or comparable ethical standards. This research has been reviewed by our research ethics committee in the Faculty of Medicine of Suez Canal University at its meeting on June 11, 2017 with reference number 3311#. Written and verbal informed consent was obtained from the selected patients.

## Availability of data and materials

The datasets used and/or analysed during the current study are available from the corresponding author on reasonable request.

## Consent for publication

We have received consent for all cases reported in our study according to an institutional consent form.

## Ethical Approval

This research was a randomized controlled trial that was carried out at the surgery departments of Suez Canal college Hospital from July 2017 to December 2018. The study was reviewed by our research ethics committee in the Faculty of Medicine of Suez Canal University at its meeting on 11/6/2017

## Funding

No funding resources.

## Author contribution

MF performed the surgery, participated in the study design and the study alignment, and drafted the manuscript and final revision.

SS Assist in the surgery and preparation of firbin glue, sequence alignment and study design, and contributed to the coordination and final revision of the manuscript.

HA participated in designing the study and surgery and helped performed the statistical analysis and draft the manuscript.

AB performed the surgical procedures and reviewed and proofread the article.

NK Prepared Autolgus fibrin glue and helped draft and prepare the final manuscript.

HF helped design the study and coordinate and critically revise the manuscript.

All authors revised and accepted the final draft for submission.

## Research Registration Unique Identifying Number (UIN)

http://www.researchregistry.com. UIN researchregistry5372.

https://www.researchregistry.com/browse-the-registry#home/registrationdetails/5e4fd67e17786100170e0538/

## Guarantor

Mohammed Faisal (Associate Professor of Surgical Oncology-Department of Surgery, Faculty of Medicine, Suez Canal University).

E-mail: m.faisal@med.suez.edu.eg.

Phone Number; 002-01226161340.

Address: Department of Surgery, Faculty of Medicine, Suez Canal University,

Circular Road, Ismailia, Egypt. Postal code: 41152

## Provenance and peer review

Not commissioned, externally peer reviewed.

## Declaration of competing interest

The authors declare no competing interest.
